# Preclinical Animal Models for Q Fever Vaccine Development

**DOI:** 10.3389/fcimb.2022.828784

**Published:** 2022-02-10

**Authors:** Mahelat Tesfamariam, Picabo Binette, Carrie Mae Long

**Affiliations:** Laboratory of Bacteriology, Division of Intramural Research, National Institute of Allergy and Infectious Diseases, National Institutes of Health, Hamilton, MT, United States

**Keywords:** *Coxiella burnetii*, Q fever, vaccine, animal modeling, guinea pig, bacterial vaccine

## Abstract

*Coxiella burnetii* is a zoonotic pathogen responsible for the human disease Q fever. While an inactivated whole cell vaccine exists for this disease, its widespread use is precluded by a post vaccination hypersensitivity response. Efforts for the development of an improved Q fever vaccine are intricately connected to the availability of appropriate animal models of human disease. Accordingly, small mammals and non-human primates have been utilized for vaccine-challenge and post vaccination hypersensitivity modeling. Here, we review the animal models historically utilized in Q fever vaccine development, describe recent advances in this area, discuss the limitations and strengths of these models, and summarize the needs and criteria for future modeling efforts. In summary, while many useful models for Q fever vaccine development exist, there remains room for growth and expansion of these models which will in turn increase our understanding of *C. burnetii* host interactions.

## Introduction

*Coxiella burnetii* is a zoonotic pathogen responsible for the human disease Q fever. Q-vax^®^, a whole cell preparation of formalin-inactivated phase I *C. burnetii*, is the only existing vaccine approved for use against Q fever. While this vaccine is highly effective at inducing long-lived immunity against *C. burnetii*, the risk of severe local reactions in individuals with pre-existing immunity (Bell et al., 1964) limits its widespread licensing and use. Intradermal skin testing was implemented in the late 1950s to assess prior exposure to *C. burnetii*, but skin testing is not completely reliable and can be expensive and cumbersome to implement (Schoffelen et al., 2014). An equally efficacious and less reactogenic vaccine is needed to eliminate the need for pre-screening and to broaden vaccination efforts in light of both natural outbreaks and biodefense applications (Long, 2021).

Animal models are invaluable tools used in the preclinical evaluation of all modern vaccine candidates. Animal models are used to evaluate vaccine safety, efficacy, route of delivery, formulation, dose, vaccine-induced immune responses, and correlates of protection (Gerdts et al., 2015). The complexity of vaccine-induced immune responses cannot currently be captured by any strategy other than the use of an intact, living system. A variety of experimental animal models have been utilized for preclinical vaccine development, with no single organism or model providing the parameters required for a streamlined process. Indeed, the most appropriate animal models vary depending on the causative agent of disease targeted by novel vaccines. For Q fever vaccine development, preclinical animal testing is particularly important, given ethical considerations precluding human *C. burnetii* challenge trials. Historically, human Q fever vaccine efficacy has been evaluated in high risk occupational groups (Marmion et al., 1984). Currently, any novel Q fever vaccine seeking United States Food and Drug Administration (FDA) approval will likely have to satisfy the conditions of the FDA Animal Rule (FDA 21CFR601.90). These conditions include the performance of rigorous animal studies in multiple predictive species resulting in well-understood mechanisms of toxicity and pharmacodynamics for dose selection. The first vaccine to be approved under this rule was the BioThrax Anthrax Vaccine Adsorbed, which received approval for a new indication in 2015 (Beasley et al., 2016). Fortuitously, several preclinical surrogate animal models have been employed for Q fever vaccine development including mice, guinea pigs, and non-human primates (Bewley, 2013). Additional species have served as models for Coxiellosis vaccine development, including livestock (Lisák, 1989) and deer (González-Barrio et al., 2017).

## Vaccine-Challenge Modeling

Vaccine-challenge models serve to evaluate the efficacy of vaccine candidates and assist in dose ranging (Golding et al., 2018). Generally, physiologically relevant routes of vaccination and infectious challenge are desired to best recapitulate eventual use of the vaccine candidate in humans. For Q fever vaccine development, ideal test systems would be comprised of physiologically relevant animal species paired with *C. burnetii* challenge *via* the aerosol route. However, models utilizing alternate species and routes of exposure are not without merit and may serve as accessible, informative model systems.

### Mice

Mice have been used in many *C. burnetii* infection and vaccination studies likely due to their size, reagent availability, and accessibility. Mice are not generally regarded as optimal models of *C. burnetii* infection due to the absence of a robust febrile response in immunocompetent strains (Theodore and Bengtson, 1942; Franti et al., 1974; Ochoa-Repáraz et al., 2007). Despite this, murine models have been used to evaluate the efficacy of various Q fever vaccine candidates, including the chloroform-methanol residue (CMR) vaccine (Waag et al., 1997) and an lipopolysaccharide peptide mimic vaccine (Peng et al., 2012).

Perhaps one of the most important considerations when using mice to model disease is strain selection. A large-scale evaluation of the susceptibility of inbred mouse strains to *C. burnetii* infection revealed several strains susceptible to clinical disease, including the BALB/c and A/J strains (Scott et al., 1987). Accordingly, the BALB/c, C57Bl/6, and A/J strains have been widely used in *C. burnetii* infection and vaccine challenge models (Bewley, 2013). Indeed, BALB/c strains generally show signs of infection when inoculated with *C. burnetii*; however, they do not display high mortality rates compared to other susceptible inbred strains (Scott et al., 1987). Thus, they have been frequently utilized in infection and vaccine-challenge studies (Scott et al., 1987; Zhang et al., 2007; Schoffelen et al., 2015; Melenotte et al., 2016). Importantly, BALB/c substrain variation may lead to divergent clinical outcomes following infection (Melenotte et al., 2016). C57Bl/6 strains are commonly favored in mechanistic studies because of the widespread ability to access congenic strains (Gregory et al., 2019). While C57Bl/6 strains typically do not exhibit overt signs of clinical illness after intraperitoneal *C. burnetii* inoculation (Scott et al., 1987), this these strains have been used in *C. burnetii* infection modeling (Leone et al., 2007) and Q fever vaccine development studies (Xiong et al., 2016; Kumaresan et al., 2021). Of all inbred strains evaluated, the A/J strain displayed the highest mortality rates when injected intraperitoneally with the virulent Nine Mile I strain of *C. burnetii* (Scott et al., 1987). Recently, Reeves, et al. utilized HLA-DR transgenic mice (B/6 background with expression of a human MHC-II allele) to robustly profile the host immune response in a vaccine-challenge model (Reeves et al., 2020). In addition to strain variation, mouse age has been recognized as a factor related to disease severity in the C57Bl/6 infection model (Leone et al., 2007). Clearly, murine strain selection and individual host factors are important considerations when this organism is used for Q fever vaccine development studies.

Additionally, route of vaccine administration and infectious challenge are both important considerations when evaluating Q fever vaccine candidates. Mice have been vaccinated *via* intraperitoneal, intratracheal, and subcutaneous routes and challenged *via* aerosol, intranasal, and intratracheal routes (Marrie et al., 1996; Waag et al., 1997; Feng et al., 2019; Gregory et al., 2019; Hu et al., 2019; Reeves et al., 2020). Bacterial distribution and tissue pathology appear to be affected by route of infectious challenge; thus, proper interpretation of experimental outcomes based on routes of vaccination and infection are important when using these models.

### Guinea Pigs

In contrast to mice, guinea pigs are known to mimic important symptoms of human *C. burnetii* infection, exhibiting a comparable dose-fever response (Benenson and Tigertt, 1956), splenomegaly, and weight loss as indicators of disease (Moos and Hackstadt, 1987; Long et al., 2019). Additionally, guinea pigs exhibit altered airway, pathological, and immunological responses to respiratory pathogens compared to mice that are generally considered to be more physiologically relevant to human disease (Gregory et al., 2019). Guinea pigs are more cumbersome and costly than mice and exhibit unique challenges regarding lack of available reagents and difficulty in performing procedures such as intratracheal instillation (Brewer and Cruise, 1997). Various guinea pig strains have been utilized in *C. burnetii* vaccine-challenge studies, with the outbred Harley strain serving as a popular model (Russell-Lodrigue et al., 2006; Fratzke et al., 2021b; Long et al., 2021).

### Non-Human Primates

Non-human primates (NHP) are important preclinical animal models as they exhibit the highest physiological and genetic similarities with humans. Limitations related to the use of NHPs include cost, ethical considerations, and containment shortcomings (Gerdts et al., 2015). Historically, macaques have served as the NHP species of choice used to model Q fever *via* aerosol inoculation (Gonder et al., 1979; Waag et al., 1999). In these studies, both cynomolgus and rhesus macaques displayed signs of clinical illness similar to that of human disease. The cynomolgus macaque model was utilized to evaluate the efficacy and immunogenicity of a CMR vaccine candidate, serving as a robust model and indicating that this vaccine could confer protection after a variety of vaccine dose and administration regimens (Waag et al., 2002). Recently, direct lower airway bacterial inoculation *via* MicroSprayer aerosolization has been introduced as a viable alternative to traditional NHP aerosol exposure techniques, producing comparable disease outcomes (Gregory et al., 2019). Additionally, the marmoset has proven to be a suitable model for studies of the pathological response to Q fever and will likely be a valuable tool for preclinical Q fever vaccine development (Nelson et al., 2020). NHP models will be particularly useful in late stages of preclinical Q fever vaccine development due to the considerations described above.

## Post vaccination Hypersensitivity Modeling

While post vaccination hypersensitivity (PVH) animal modeling has been crucial in the evaluation of emerging Q fever vaccine candidates, both the availability of diverse models and the understanding of immunological mechanisms underlying the PVH response have remained largely elusive. Severe local reactions in pre-immune individuals were first observed in the early 1950s when Q fever vaccination programs were initially implemented (Meiklejohn and Lennette, 1950; Bell et al., 1964). A skin testing regime was adopted to screen individuals with pre-existing immunity to *C. burnetii* and was further refined as new methods for formulating the vaccine became available in the early 1960s (Lackman et al., 1962; Ormsbee, 1962; Luoto et al., 1963).

### Early History

Following the development of novel methods of vaccine purification and bacterial fractionation, the human Q fever skin test was adapted to sensitized rabbits (Anacker et al., 1962). Rabbits sensitized *via* intradermal injection of 50 µg phase I *C. burnetii* whole cell vaccine (WCV) were skin tested intradermally with increasing doses of different fractions of *C. burnetii*, ranging from 0.02 to 10 µg, to determine the minimal dose needed to recapitulate the skin reaction observed in sensitized humans. This model was also used to evaluate the reactogenicity of various bacterial fractions. While unsensitized rabbits experienced lesions, these typically did not occur until at least 72 hours post skin testing and at much larger doses (31.1x) than that of the sensitized animals. The responses in the sensitized rabbits were hypothesized to be delayed-type hypersensitivity (DTH) responses, but this was not conclusively evaluated. No major modifications to the rabbit model have been made since these studies.

### Guinea Pigs

Ascher et al. introduced the first validated guinea pig PVH model in 1983 (Ascher et al., 1983a). Here, Hartley guinea pigs were sensitized *via* immunization with a 50 μg dose of phase I WCV emulsified in either Freund’s complete (QFA) or incomplete adjuvant (IFA) divided into the four footpads and skin tested with a 60 ng dose of the vaccine two six weeks post sensitization in a shaved flank. While skin test depth was not described, the photomicrographs of skin test sites demonstrated reactions in the deep dermis or subcutis, indicating that subcutaneous injections were employed (Wilhelmsen and Waag, 2000). Because data from rabbit models suggested that the PVH reaction could be a DTH response, prior to skin testing, Ascher et al. treated a group of guinea pigs sensitized with WCV with cyclophosphamide, which had previously been shown to enhance DTH responses (Ascher et al., 1977). As hypothesized, the cyclophosphamide-pretreated, sensitized guinea pigs experienced significantly increased reactions, as quantified by increases in skin thickness, further supporting the PVH-DTH hypothesis. Consistent with past human studies, this guinea pig model yielded data suggestive of granulatomous DTH (Ascher et al., 1983b).

Since introduced for PVH, the guinea pig model has remained the gold standard for vaccine reactogenicity studies (Bewley, 2013). Due to undesirable side effects of footpad inoculation, Ruble et al. sensitized hairless Hartley guinea pigs either subcutaneously (SC) or intradermally (ID) with a total of 60 µg WCV or CMR with or without IFA, distributed over numerous injection sites (Ruble et al., 1994). Six weeks later, animals were subcutaneously skin tested with vaccine doses ranging from 60 to 6,000 ng. While human skin tests are performed ID, SC injection was tested as an alternative because the vaccine is administered SC and would therefore more closely mimic the PVH reaction in pre-immune individuals. Hairless Hartley guinea pigs gave comparable results to prior models, with peak induration at 9 days (Ascher et al., 1983a). Using the hairless strain was advantageous as it reduced any additional irritation caused by shaving that could ultimately interfere with interpretation of the skin test response.

Wilhelmsen and Waag evaluated aerosol and intraperitoneal inoculation as alternative sensitization routes to SC vaccination in Hartley guinea pigs (Wilhelmsen and Waag, 2000). A 30 µg sensitization dose was used which was a substantial departure from the maximal 60 ng doses used in prior studies. As in previous studies, induration was greatest at 8-12 days post skin testing.

The latest refinements to the guinea pig model came from a 2018 study performed by Baeten et al. which aimed to standardize the guinea pig PVH model (Baeten et al., 2018). This study further explored intranasal and intraperitoneal sensitization routes and evaluated differences between skin tests performed either intradermally or subcutaneously at different time points post sensitization using Coxevac^®^, the ruminant vaccine for Q fever formulated from the NMI strain of *C. burnetii*. Recently, similar guinea pig models have been used to evaluate the reactogenicity of several Q fever vaccine candidates, including a genetically modified WCV (Long et al., 2021), exemplifying the utility of this model. Cumulatively, the data collected from guinea pigs suggest that PVH reactions are likely dominated by a granulatomous DTH reaction in this species, which is mediated by memory T cells producing Th1 cytokines (Kobayashi et al., 2001).

### Mice

While the guinea pig reactogenicity model has been useful determining the reactogenicity of vaccine candidates, further characterization of the PVH response has been severely limited due to a lack of experimental reagents available for guinea pigs. Therefore, development of model systems for which there are more reagents readily available should be considered as elucidation of the mechanisms underlying these responses will provide insight for the development of novel Q fever vaccines. The first use of mice in PVH modeling was performed by Kazár et al. in 1987 (Kazár et al., 1987). In this study, mice were sensitized with 100 µg of phase I *C. burnetii* intraperitoneally and mice were skin tested *via* hind footpad injection of WCV and CMR (30-0.003 µg). Mice elicited DTH responses, but at a higher minimum dose (0.3 µg) compared to rabbits and guinea pigs (0.03 µg). Recently, Fratzke et al. tested three different mice strains (SKHI, C57Bl/6 and BALBc) for their ability to recapitulate the DTH response when sensitized, proposing the SKH1 and C57Bl/6 strains as viable options for this purpose (Fratzke et al., 2021a). Currently, guinea pigs are the primary model for assessing Q fever vaccine reactogenicity, but mice are an exciting complementary option. Important considerations regarding PVH modeling are dose selection, skin testing injection depth, sensitization method (e.g. infection vs vaccination), and species selection.

## Conclusions and Outlook

**Figure 1:**
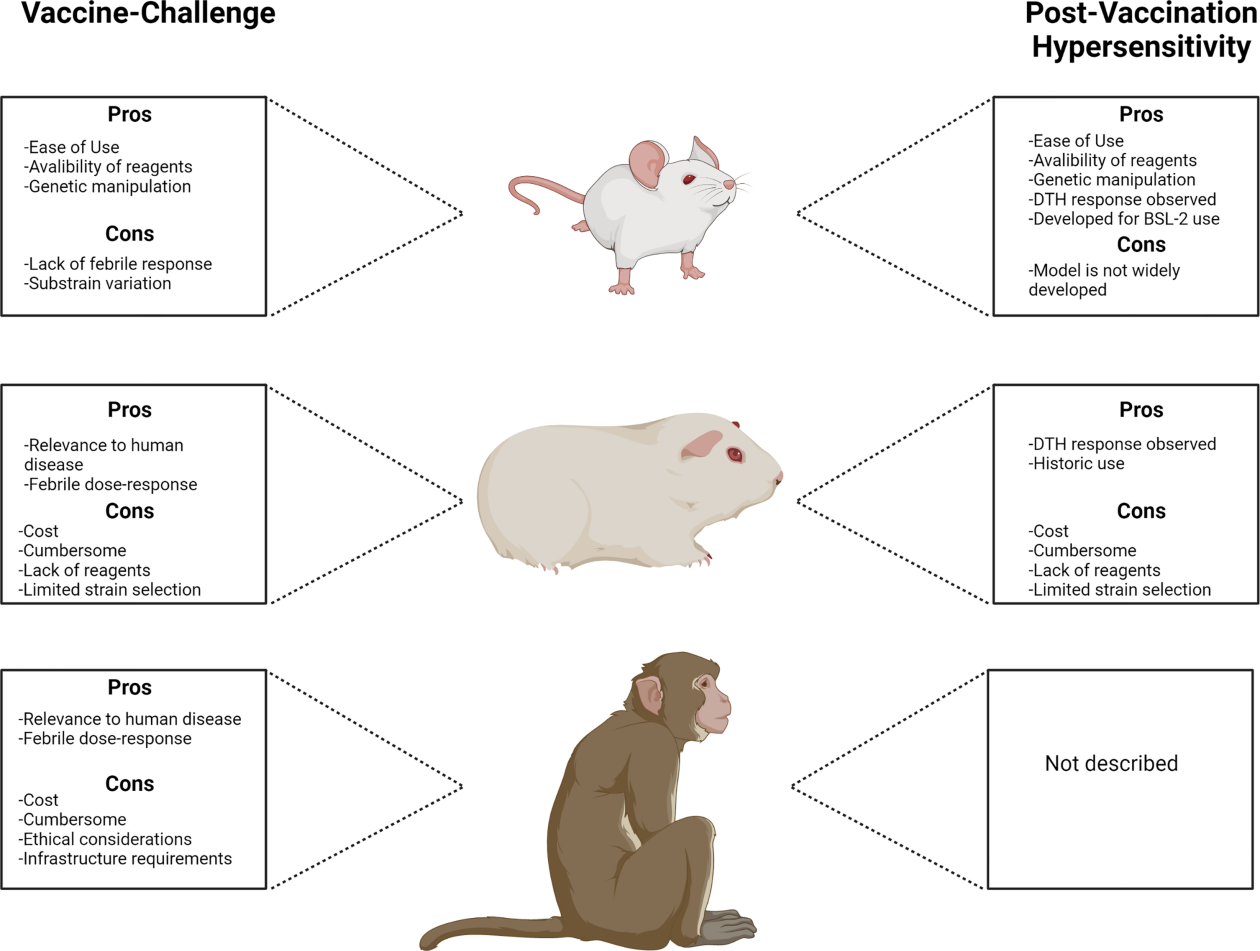
Comparison of animal models used for Q fever vaccine development with principal advantages and disadvantages associated with each model.

Ultimately, the quality of a preclinical animal model can be assessed based on its physiologic relevance to human disease. Relevant physiologic characteristics may include redundancy in anatomy and components of the immune system, clinical response to infection, pathogenesis, and duration of biologic responses (Gerdts et al., 2015). Translating preclinical data to human vaccine trials and eventual deployment remains a major challenge but may be addressed by using highly physiologically relevant animal models paired with an understanding of the strengths and limitations of the models chosen. For *C. burnetii* vaccine-challenge and PVH modeling, several species have proven their usefulness in the studies described above and exhibit unique advantages and limitations (**Figure 1**). Guinea pigs appear to be the premier small mammal model of Q fever due to their robust physiologic relevance to human disease. NHPs will likely serve as valuable model systems for Q fever vaccine development but their accessibility precludes widespread use, particularly in early preclinical vaccine development. PVH modeling is dominated by the guinea pig but mice appear to be a promising complementary model for mechanistic evaluation of this response. Recently, a larval *Galleria mellonella* model of *C. burnetii* infection was introduced by Norville, et al. (Norville et al., 2014). This advance introduces the possibility of the use of non-mammalian models in future vaccine development efforts. Perhaps species such as *G. mellonella* or even those with more developed immune systems such as *Danio rerio* (zebrafish) will provide accessible pathways to complement existing mammalian models. Indeed, zebrafish have proven useful for preliminary human vaccine safety screening (Bailone et al., 2020). We feel that expansion of all relevant *C. burnetii* infection, vaccination, and PVH models will increase knowledge of correlates of protection and potential screening biomarkers, serving as valuable tools not only for vaccine development but to increase general knowledge of these important host-pathogen interactions.

Ultimately, we suggest that there is no “perfect model” for Q fever vaccine development. Indeed, the complexity of relevant biologic responses, the challenges of working with *C. burnetii*, and the lack of data regarding human responses to vaccination and infection are a few of the factors contributing to this quandary. Regardless, exciting progress has recently been made in *C. burnetii* host-pathogen animal modeling. The expansion of these models and ensuing comparative analysis will aid in the development of an improved Q fever vaccine by providing the most relevant and useful models possible. For example, it will be crucial to utilize these models to further elucidate the PVH response and relate this to the human condition. Considering that the current QVax^®^ skin test regimen is contained within a 7 day window (Sellens et al., 2018) and prior reports indicate that this test can be read as early as 48 hours post skin testing (Lackman et al., 1962), the possible involvement of a tuberculin-type DTH component in the human PVH response, with clinical symptoms manifesting earlier than that of granulomatous DTH, should be considered during model development. Additional considerations for the field include the influence of biological sex and age on choice of model and Q fever vaccine efficacy. Considerations such as these serve to more wholly represent the human population affected by disease and eventual vaccination. These two factors are known to play a significant role in infectious disease pathogenesis (Bijkerk et al., 2010; Ingersoll, 2017) and vaccine efficacy (Lord, 2013; Fink et al., 2018; Bignucolo et al., 2021). Specifically, they appear to influence similar outcomes related to *C. burnetii* host-pathogen interactions (Franti et al., 1974; Leone et al., 2004; Textoris et al., 2010). With more representative and comprehensive animal models of disease, our vaccine development efforts will thrive for *C. burnetii* and beyond.

## Author Contributions

MT, PB, and CL conceptualized, wrote, and edited the manuscript. All authors contributed to the article and approved the submitted version.

## Funding

This work was supported by the Intramural Research Program of the National Institutes of Health, National Institute of Allergy and Infectious Diseases (ZIAAI001331).

## Conflict of Interest

The authors declare that the research was conducted in the absence of any commercial or financial relationships that could be construed as a potential conflict of interest.

## Publisher’s Note

All claims expressed in this article are solely those of the authors and do not necessarily represent those of their affiliated organizations, or those of the publisher, the editors and the reviewers. Any product that may be evaluated in this article, or claim that may be made by its manufacturer, is not guaranteed or endorsed by the publisher.

## References

[B1] Anacker R. L. Lackman D. B. Pickens E. G. Ribi E. (1962). Antigenic and Skin-Reactive Properties of Fractions of Coxiella Burnetii. J. Immunol. 89, 145.

[B2] Ascher M. S. Berman M. A. Parker D. Turk J. L. (1983a). Experimental Model for Dermal Granulomatous Hypersensitivity in Q Fever. Infect. Immun. 39, 388–393. doi: 10.1128/iai.39.1.388-393.1983 6822420 PMC347951

[B3] Ascher M. S. Berman M. A. Ruppanner R. (1983b). Initial Clinical and Immunologic Evaluation of a New Phase I Q Fever Vaccine and Skin Test in Humans. J. Infect. Dis. 148, 214–222. doi: 10.1093/infdis/148.2.214 6350491

[B4] Ascher M. S. Jahrling D. Gm. Harrington D. G. Kishimoto R. A. McGann V. g. (1980). Mechanisms of Protective Immunogenicity of Microbial Vaccines: Effects of Cyclophosphamide Pretreatment in Venezuelan Encephalitis, Q Fever, and Tularemia. Clin. Exp. Immunol. 41 (2), 225–236–323. 7438552 PMC1536999

[B5] Baeten L. A. Podell B. K. Sluder A. E. Garritsen A. Bowen R. A. Poznansky M. C. (2018). Standardized Guinea Pig Model for Q Fever Vaccine Reactogenicity. PloS One 13, e0205882. doi: 10.1371/journal.pone.0205882 30312355 PMC6185858

[B6] Bailone R. L. Fukushima H. C. S. Ventura Fernandes B. H. De Aguiar L. K. Corrêa T. Janke H.. (2020). Zebrafish as an Alternative Animal Model in Human and Animal Vaccination Research. Lab. Anim. Res. 36, 13–13. doi: 10.1186/s42826-020-00042-4 32382525 PMC7203993

[B7] Beasley D. W. C. Brasel T. L. Comer J. E. (2016). First Vaccine Approval Under the FDA Animal Rule. NPJ Vaccines 1, 16013. doi: 10.1038/npjvaccines.2016.13 29263855 PMC5707879

[B8] Bell J. F. Lackman D. B. Meis A. Hadlow W. J. (1964). Recurrent Reaction of Site of Q Fever Vaccination in a Sensitized Person. Mil. Med. 129, 591–595. doi: 10.1093/milmed/129.7.591 14199980

[B9] Benenson A. S. Tigertt W. D. (1956). Studies on Q Fever in Man. Trans. Assoc. Am. Physicians 69, 98–104. 13380951

[B10] Bewley K. R. (2013). Animal Models of Q Fever (*Coxiella Burnetii*). Comp. Med. 63, 469–476. 24326221 PMC3866982

[B11] Bignucolo A. Scarabel L. Mezzalira S. Polesel J. Cecchin E. Toffoli G. (2021). Sex Disparities in Efficacy in COVID-19 Vaccines: A Systematic Review and Meta-Analysis. Vaccines 9, 825. doi: 10.3390/vaccines9080825 34451950 PMC8402482

[B12] Bijkerk P. Van Lier E. A. Van Vliet J. A. Kretzschmar M. E. (2010). Effects of Ageing on Infectious Disease. Ned. Tijdschr Geneeskd. 154, A1613. 20977793

[B13] Brewer N. R. Cruise L. J. (1997). The Respiratory System of the Guinea Pig: Emphasis on Species Differences. Contemp. Top. Lab. Anim. Sci. 36, 100–108. 12456198

[B14] Feng J. Hu X. Fu M. Dai L. Yu Y. Luo W.. (2019). Enhanced Protection Against Q Fever in BALB/C Mice Elicited by Immunization of Chloroform-Methanol Residue of *Coxiella Burnetii via* Intratracheal Inoculation. Vaccine 37, 6076–6084. doi: 10.1016/j.vaccine.2019.08.041 31477436

[B15] Fink A. L. Engle K. Ursin R. L. Tang W.-Y. Klein S. L. (2018). Biological Sex Affects Vaccine Efficacy and Protection Against Influenza in Mice. Proc. Natl. Acad. Sci. 115, 12477–12482. doi: 10.1073/pnas.1805268115 30455317 PMC6298067

[B16] Franti C. E. Behymer D. E. Goggin J. E. Wright M. E. (1974). Splenomegaly, Sex, and Other Characteristics of Laboratory Animals Used for Primary Isolations of *Coxiella Burnetii* . Lab. Anim. Sci. 24, 656–665. 4369207

[B17] Fratzke A. P. Jan S. Felgner J. Liang L. Nakajima R. Jasinskas A.. (2021b). Subunit Vaccines Using TLR Triagonist Combination Adjuvants Provide Protection Against *Coxiella Burnetii* While Minimizing Reactogenic Responses. Front. Immunol. 12. doi: 10.3389/fimmu.2021.653092 PMC8010241 33815413

[B18] Fratzke A. P. Gregory A. E. Van Schaik E. J. Samuel J. E. (2021a). *Coxiella Burnetii* Whole Cell Vaccine Produces a Th1 Delayed-Type Hypersensitivity Response in a Novel Sensitized Mouse Model. Front. Immunol. 12, 754712. doi: 10.3389/fimmu.2021.754712 34616410 PMC8488435

[B19] Gerdts V. Wilson H. L. Meurens F. Van Drunen Littel-Van Den Hurk S. Wilson D. Walker S.. (2015). Large Animal Models for Vaccine Development and Testing. Ilar. J. 56, 53–62. doi: 10.1093/ilar/ilv009 25991698

[B20] Golding H. Khurana S. Zaitseva M. (2018). What is the Predictive Value of Animal Models for Vaccine Efficacy in Humans? The Importance of Bridging Studies and Species-Independent Correlates of Protection. Cold Spring Harbor Perspect. Biol. 10, a028902. doi: 10.1101/cshperspect.a028902 PMC5880166 28348035

[B21] Gonder J. C. Kishimoto R. A. Kastello M. D. Pedersen C. E. Jr. Larson E. W. (1979). Cynomolgus Monkey Model for Experimental Q Fever Infection. J. Infect. Dis. 139, 191–196. doi: 10.1093/infdis/139.2.191 108342

[B22] González-Barrio D. Ortiz J. A. Ruiz-Fons F. (2017). Estimating the Efficacy of a Commercial Phase I Inactivated Vaccine in Decreasing the Prevalence of Coxiella Burnetii Infection and Shedding in Red Deer (Cervus Elaphus). Front. Veterinary Sci. 4. 10.3389/fvets.2017.00208 PMC5723644 29270411

[B23] Gregory A. E. Van Schaik E. J. Russell-Lodrigue K. E. Fratzke A. P. Samuel J. E. (2019). *Coxiella Burnetii* Intratracheal Aerosol Infection Model in Mice, Guinea Pigs, and Nonhuman Primates. Infect. Immun. 87, e00178–19. doi: 10.1128/IAI.00178-19 PMC6867829 31501249

[B24] Hu X. Yu Y. Feng J. Fu M. Dai L. Lu Z.. (2019). Pathologic Changes and Immune Responses Against *Coxiella Burnetii* in Mice Following Infection *via* non-Invasive Intratracheal Inoculation. PloS One 14, e0225671. doi: 10.1371/journal.pone.0225671 31805090 PMC6894818

[B25] Ingersoll M. A. (2017). Sex Differences Shape the Response to Infectious Diseases. PloS Pathog. 13, e1006688-e1006688. doi: 10.1371/journal.ppat.1006688 29284060 PMC5746274

[B26] Kazár J. Schramek S. Lisák V. Brezina R. (1987). Antigenicity of Chloroform-Methanol-Treated *Coxiella Burnetii* Preparations. Acta Virol. 31, 158–167. 2886025

[B27] Kobayashi K. Kaneda K. Kasama T. (2001). Immunopathogenesis of Delayed-Type Hypersensitivity. Microsc. Res. Tech 53, 241–245. doi: 10.1002/jemt.1090 11340669

[B28] Kumaresan V. Alam S. Zhang Y. Zhang G. (2021). The Feasibility of Using *Coxiella Burnetii* Avirulent Nine Mile Phase II Viable Bacteria as a Live Attenuated Vaccine Against Q Fever. Front. Immunol. 12. doi: 10.3389/fimmu.2021.754690 PMC8594375 34795669

[B29] Lackman D. B. Bell E. J. Bell J. F. Pickens E. G. (1962). Intradermal Sensitivity Testing in Man With a Purified Vaccine for Q Fever. Am. J. Public Health Nations Health 52, 87–93. doi: 10.2105/AJPH.52.1.87 14461400 PMC1522671

[B30] Leone M. Bechah Y. Meghari S. Lepidi H. Capo C. Raoult D.. (2007). *Coxiella Burnetii* Infection in C57BL/6 Mice Aged 1 or 14 Months. FEMS Immunol. Med. Microbiol. 50, 396–400. doi: 10.1111/j.1574-695X.2007.00272.x 17555529

[B31] Leone M. Honstettre A. Lepidi H. Capo C. Bayard F. Raoult D.. (2004). Effect of Sex on *Coxiella Burnetii* Infection: Protective Role of 17beta-Estradiol. J. Infect. Dis. 189, 339–345. doi: 10.1086/380798 14722900

[B32] Lisák V. (1989). Experience in Vaccinating Farm Animals for Preventing Q Fever in Humans. Tr Inst. Im Pastera 66 143-153, 174. 2485300

[B33] Long C. M. (2021). Q Fever Vaccine Development: Current Strategies and Future Considerations. Pathogens 10, 1223. doi: 10.3390/pathogens10101223 34684172 PMC8539696

[B34] Long C. M. Beare P. A. Cockrell D. C. Fintzi J. Tesfamariam M. Shaia C. I.. (2021). Contributions of Lipopolysaccharide and the Type IVB Secretion System to *Coxiella Burnetii* Vaccine Efficacy and Reactogenicity. NPJ Vaccines 6, 38. doi: 10.1038/s41541-021-00296-6 33741986 PMC7979919

[B35] Long C. M. Beare P. A. Cockrell D. C. Larson C. L. Heinzen R. A. (2019). Comparative Virulence of Diverse *Coxiella Burnetii* Strains. Virulence 10, 133–150. doi: 10.1080/21505594.2019.1575715 30782062 PMC6389282

[B36] Lord J. M. (2013). The Effect of Ageing of the Immune System on Vaccination Responses. Hum. Vaccines Immunotherapeutics 9, 1364–1367. doi: 10.4161/hv.24696 PMC3901832 23584248

[B37] Luoto L. Bell J. F. Casey M. Lackman D. B. (1963). Q Fever Vaccination of Human Volunteers. I. The Serologic and Skin-Test Response Following Subcutaneous Injections. Am. J. Hyg. 78, 1–15. doi: 10.1093/oxfordjournals.aje.a120324 14043544

[B38] Marmion B. P. Ormsbee R. A. Kyrkou M. Wright J. Worswick D. Cameron S.. (1984). Vaccine Prophylaxis of Abattoir-Associated Q Fever. Lancet 2, 1411–1414. doi: 10.1016/S0140-6736(84)91617-9 6151039

[B39] Marrie T. J. Stein A. Janigan D. Raoult D. (1996). Route of Infection Determines the Clinical Manifestations of Acute Q Fever. J. Infect. Dis. 173, 484–487. doi: 10.1093/infdis/173.2.484 8568318

[B40] Meiklejohn G. Lennette E. H. (1950). Q Fever in California. I. Observations on Vaccination of Human Beings. Am. J. Hyg. 52, 54–64. 15432441

[B41] Melenotte C. Lepidi H. Nappez C. Bechah Y. Audoly G. Terras J.. (2016). Mouse Model of *Coxiella Burnetii* Aerosolization. Infect. Immun. 84, 2116–2123. doi: 10.1128/IAI.00108-16 27160294 PMC4936361

[B42] Moos A. Hackstadt T. (1987). Comparative Virulence of Intra- and Interstrain Lipopolysaccharide Variants of *Coxiella Burnetii* in the Guinea Pig Model. Infect. Immun. 55, 1144–1150. doi: 10.1128/iai.55.5.1144-1150.1987 3570458 PMC260482

[B43] Nelson M. Salguero F. J. Hunter L. Atkins T. P. (2020). A Novel Marmoset (Callithrix Jacchus) Model of Human Inhalational Q Fever. Front. Cell Infect. Microbiol. 10, 621635. doi: 10.3389/fcimb.2020.621635 33585288 PMC7876459

[B44] Norville I. H. Hartley M. G. Martinez E. Cantet F. Bonazzi M. Atkins T. P. (2014). Galleria Mellonella as an Alternative Model of *Coxiella Burnetii* Infection. Microbiology 160, 1175–1181. doi: 10.1099/mic.0.077230-0 24677067

[B45] Ochoa-Repáraz J. Sentissi J. Trunkle T. Riccardi C. Pascual D. W. (2007). Attenuated *Coxiella Burnetii* Phase II Causes a Febrile Response in Gamma Interferon Knockout and Toll-Like Receptor 2 Knockout Mice and Protects Against Reinfection. Infect. Immun. 75, 5845–5858. doi: 10.1128/IAI.00901-07 17893129 PMC2168348

[B46] Ormsbee R. A. (1962). A Method of Purifying *Coxiella Burnetii* and Other Pathogenic Rickettsiae. J. Immunol. 88, 100–108. 14482336

[B47] Peng Y. Zhang Y. Mitchell W. J. Zhang G. (2012). Development of a Lipopolysaccharide-Targeted Peptide Mimic Vaccine Against Q Fever. J. Immunol. 189, 4909–4920. doi: 10.4049/jimmunol.1201622 23053512 PMC3833726

[B48] Reeves P. M. Raju Paul S. Baeten L. Korek S. E. Yi Y. Hess J.. (2020). Novel Multiparameter Correlates of *Coxiella Burnetii* Infection and Vaccination Identified by Longitudinal Deep Immune Profiling. Sci. Rep. 10. doi: 10.1038/s41598-020-69327-x PMC7414860 32770104

[B49] Ruble D. L. Elliott J. J. Waag D. M. Jaax G. P. (1994). A Refined Guinea Pig Model for Evaluating Delayed-Type Hypersensitivity Reactions Caused by Q Fever Vaccines. Lab. Anim. Sci. 44, 608–612. 7898035

[B50] Russell-Lodrigue K. E. Zhang G. Q. Mcmurray D. N. Samuel J. E. (2006). Clinical and Pathologic Changes in a Guinea Pig Aerosol Challenge Model of Acute Q Fever. Infect. Immun. 74, 6085–91. doi: 10.1128/IAI.00763-06 PMC1695512 17057087

[B51] Schoffelen T. Herremans T. Sprong T. Nabuurs-Franssen M. van der Meer J. W. Joosten L. A.. (2014). Immunogenicity of the Q Fever Skin Test. J. Infect. 69, 161–164. doi: 10.1016/j.jinf.2014.03.008 24642208

[B52] Schoffelen T. Self J. S. Fitzpatrick K. A. Netea M. G. Van Deuren M. Joosten L. A.. (2015). Early Cytokine and Antibody Responses Against *Coxiella Burnetii* in Aerosol Infection of BALB/C Mice. Diagn. Microbiol. Infect. Dis. 81, 234–239. doi: 10.1016/j.diagmicrobio.2014.12.008 25618420 PMC4740919

[B53] Scott G. H. Williams J. C. Stephenson E. H. (1987). Animal Models in Q Fever: Pathological Responses of Inbred Mice to Phase I *Coxiella Burnetii* . Microbiology 133, 691–700. doi: 10.1099/00221287-133-3-691 3655728

[B54] Sellens E. Bosward K. L. Willis S. Heller J. Cobbold R. Comeau J. L.. (2018). Frequency of Adverse Events Following Q Fever Immunisation in Young Adults. Vaccines (Basel) 6. doi: 10.3390/vaccines6040083 PMC6313871 30551615

[B55] Textoris J. Ban L. H. Capo C. Raoult D. Leone M. Mege J.-L. (2010). Sex-Related Differences in Gene Expression Following *Coxiella Burnetii* Infection in Mice: Potential Role of Circadian Rhythm. PloS One 5, e12190. doi: 10.1371/journal.pone.0012190 20730052 PMC2921390

[B56] Theodore L. P. Bengtson I. A. (1942). The Histopathology of Experimental “Q” Fever in Mice. Public Health Rep. (1896-1970) 57, 790–798. doi: 10.2307/4584109

[B57] Waag D. M. Byrne W. R. Estep J. Gibbs P. Pitt M. L. Banfield C. M. (1999). Evaluation of Cynomolgus (Macaca Fascicularis) and Rhesus (Macaca Mulatta) Monkeys as Experimental Models of Acute Q Fever After Aerosol Exposure to Phase-I *Coxiella Burnetii* . Lab. Anim. Sci. 49, 634–638. 10638499

[B58] Waag D. M. England M. J. Pitt M. L. (1997). Comparative Efficacy of a *Coxiella Burnetii* Chloroform:Methanol Residue (CMR) Vaccine and a Licensed Cellular Vaccine (Q-Vax) in Rodents Challenged by Aerosol. Vaccine 15, 1779–1783. doi: 10.1016/S0264-410X(97)00107-2 9364683

[B59] Waag D. M. England M. J. Tammariello R. F. Byrne W. R. Gibbs P. Banfield C. M.. (2002). Comparative Efficacy and Immunogenicity of Q Fever Chloroform:Methanol Residue (CMR) and Phase I Cellular (Q-Vax) Vaccines in Cynomolgus Monkeys Challenged by Aerosol. Vaccine 20, 2623–2634. doi: 10.1016/S0264-410X(02)00176-7 12057622

[B60] Wilhelmsen C. L. Waag D. M. (2000). Guinea Pig Abscess/Hypersensitivity Model for Study of Adverse Vaccination Reactions Induced by Use of Q Fever Vaccines. Comp. Med. 50, 374–378. 11020154

[B61] Xiong X. Jiao J. Gregory A. E. Wang P. Bi Y. Wang X.. (2016). Identification of *Coxiella Burnetii* CD8+ T-Cell Epitopes and Delivery by Attenuated Listeria Monocytogenes as a Vaccine Vector in a C57BL/6 Mouse Model. J. Infect. Dis. 215, 1580–1589. doi: 10.1093/infdis/jiw470 PMC6281342 27703037

[B62] Zhang G. Russell-Lodrigue K. E. Andoh M. Zhang Y. Hendrix L. R. Samuel J. E. (2007). Mechanisms of Vaccine-Induced Protective Immunity Against *Coxiella Burnetii* Infection in BALB/C Mice. J. Immunol. 179, 8372–8380. doi: 10.4049/jimmunol.179.12.8372 18056383

